# MDM2/X inhibitors under clinical evaluation: perspectives for the management of hematological malignancies and pediatric cancer

**DOI:** 10.1186/s13045-017-0500-5

**Published:** 2017-07-03

**Authors:** Veronica Tisato, Rebecca Voltan, Arianna Gonelli, Paola Secchiero, Giorgio Zauli

**Affiliations:** Department of Morphology, Surgery and Experimental Medicine and LTTA Centre, University of Ferrara, Via Fossato di Mortara 66, 44121 Ferrara, Italy

**Keywords:** MDM2, MDMX, Pharmacological inhibitor, Clinical studies, Leukemia, Pediatric tumors

## Abstract

The two murine double minute (MDM) family members MDM2 and MDMX are at the center of an intense clinical assessment as molecular target for the management of cancer. Indeed, the two proteins act as regulators of P53, a well-known key controller of the cell cycle regulation and cell proliferation that, when altered, plays a direct role on cancer development and progression. Several evidence demonstrated that functional aberrations of P53 in tumors are in most cases the consequence of alterations on the MDM2 and MDMX regulatory proteins, in particular in patients with hematological malignancies where *TP53* shows a relatively low frequency of mutation while *MDM2* and *MDMX* are frequently found amplified/overexpressed. The pharmacological targeting of these two P53-regulators in order to restore or increase P53 expression and activity represents therefore a strategy for cancer therapy. From the discovery of the Nutlins in 2004, several compounds have been developed and reported with the ability of targeting the P53-MDM2/X axis by inhibiting MDM2 and/or MDMX. From natural compounds up to small molecules and stapled peptides, these MDM2/X pharmacological inhibitors have been extensively studied, revealing different biological features and different rate of efficacy when tested in in vitro and in vivo experimental tumor models. The data/evidence coming from the preclinical experimentation have allowed the identification of the most promising molecules and the setting of clinical studies for their evaluation as monotherapy or in therapeutic combination with conventional chemotherapy or with innovative therapeutic protocols in different tumor settings. Preliminary results have been recently published reporting data about safety, tolerability, potential side effects, and efficacy of such therapeutic approaches. In this light, the aim of this review is to give an updated overview about the state of the art of the clinical evaluation of MDM2/X inhibitor compounds with a special attention to hematological malignancies and to the potential for the management of pediatric cancers.

## Background

### The MDM2/X genes and proteins

The murine double minute 2 (*MDM2*) gene was originally discovered through screening of a cDNA library of RNA isolated from a spontaneously transformed derivative of mouse 3T3 cells [1]. The characterization of the newly discovered *MDM2* gene showed its tumorigenic potential when experimentally overexpressed, a preliminary evidence followed by the full identification and the structural description of a key evolutionarily conserved gene with a crucial role in regulating cellular growth [2, 3]. Human *MDM2* gene (*HDM2*) maps on chromosome 12q14.3–q15 and both expression and functions are tightly regulated at transcriptional level (e.g., presence of different promoters, gene polymorphisms, splicing variants), at translational level and at post-translational level by several cell signals that regulate protein accumulation and activity [4]. The *MDM2* gene codifies for an E3 ubiquitin ligase that acts as a powerful inhibitor of the tumor suppressor P53, the master regulator of the cell cycle progression, in a feedback loop that is attracting great interests and attention as a potential target for tumor therapeutic purposes [5]. The tight link between MDM2 and P53 has been recently addressed from Tan and colleagues in an interesting analysis of the evolutionary history of the *MDM* genes in relation to *P53* [6]. In their study, the authors have highlighted a preserved cellular role throughout history of this genes’ family because, as expected, the maintenance of DNA integrity and the ability to respond to DNA damage are inalienable functions from an evolutionary perspective. Of note, the functional relationship between MDM and P53 has been found also in organisms that existed for one billion years, suggesting that these two proteins have evolved together to maintain preserved and controlled synergic functions [6]. However, although the interest on MDM2 rises on its key biological target, MDM2 can also interact with other molecules including upstream regulators (effectors) and downstream proteins (affectors) leading to several P53-independent effects [7].

**Fig. 1:**
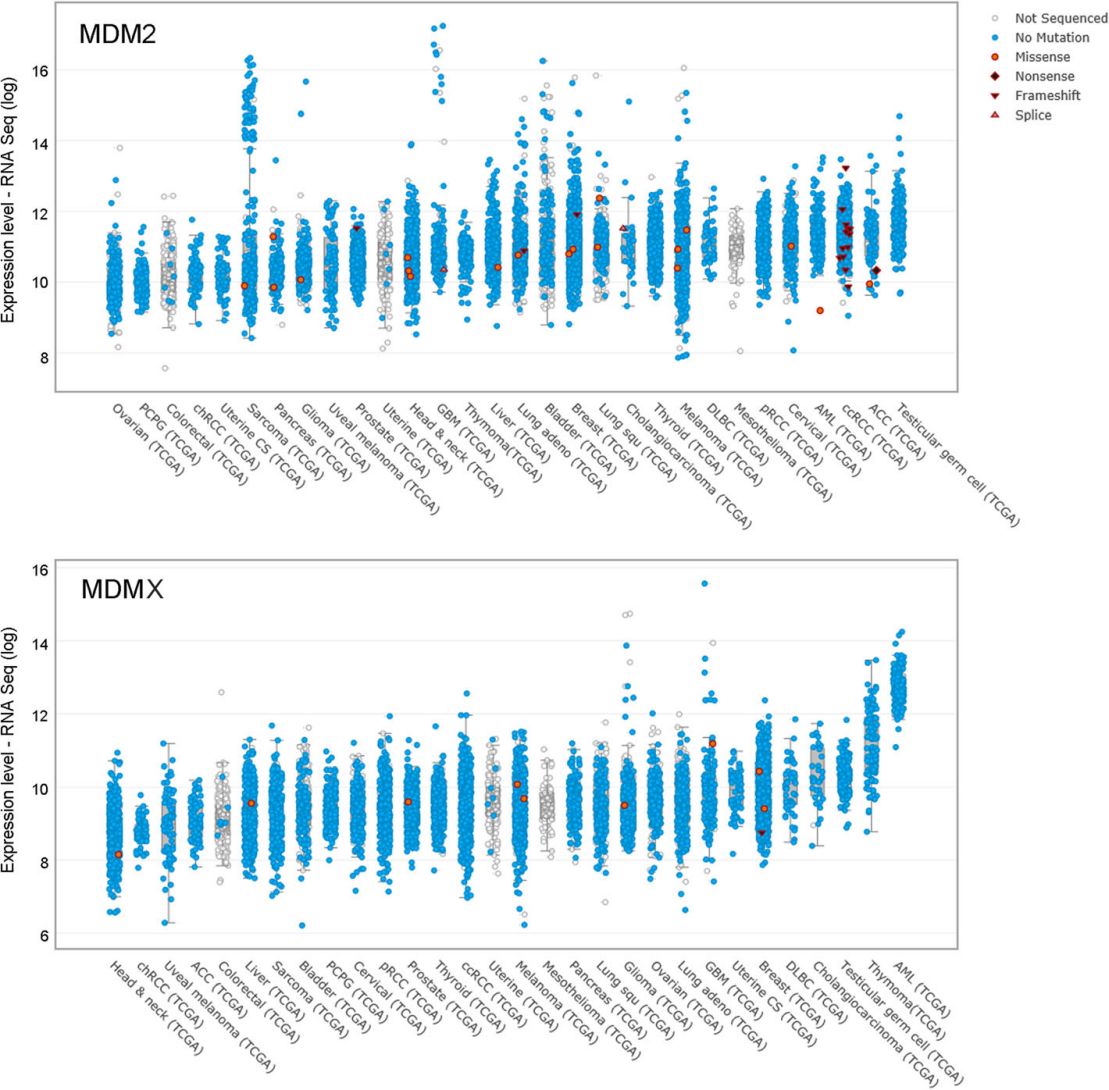
Expression levels of MDM2 and MDMX in human cancers. Graphical representation of the expression levels of MDM2 (*upper panel*) and MDMX (*lower panel*) in human cancers obtained from studies collected in cBioPortal For Cancer Genomics (www.cbioportal.org). The picture shows RNA values of patients expressing the wild-type or mutated genes. *TCGA* The Cancer Genome Atlas Network

The tumor suppressor P53 has been defined as “guardian of the genome” in light of its nature, a multifunctional transcription factor that can be activated by several stress signals and which is able to regulate a wide panel of target genes leading to different biological functions, overall aiming at monitoring and controlling cell cycle progression and cellular proliferation state [8–10]. The biological impact of any alteration of *TP53* gene and/or P53 protein activity can be summarized by the evidence that *TP53* knockout mice appear normal but are predisposed to spontaneous development of tumors at young age [11, 12], and that the presence of mutant TP53 allele makes the mice more prone to the development of tumors [13]. In humans, the protein is mutated in most of solid tumors [14] and it has been widely demonstrated that the newly acquired oncogenic functions allow uncontrolled cell survival/proliferation and acquisition of invasive/metastatic potentials [15]. In the context of hematological malignancies, the frequency of mutations of *TP53* has a minor impact compared to solid tumors since most types of leukemia express wild-type P53, and the reported functional aberrations of the protein are in most cases the consequence of alterations on P53 regulatory proteins, in particular MDM2 and its paralog MDMX. The *MDMX* (namely *MDM4*) gene is located on human chromosome 1q32 and encodes for an MDM2 effector that plays a key role in both modulating P53 and regulating MDM2 activity. This protein was discovered more than 20 years ago as an additional P53-modulating factor characterized by a structural similarity to MDM2 [16], showing a stimulatory effect on the E3 activity of MDM2 without a detectable direct E3 ubiquitin ligase activity of MDMX itself [17]. MDM2 and MDMX are therefore key (non-overlapping) regulators of P53 as demonstrated by some fundamental in vivo preclinical experiments performed in *MDM2* and *MDMX* null mice, showing that both *MDM2* ^*−/−*^ and *MDMX* ^*−/−*^ genetic background are characterized by embryonic lethality and that the lethality can be completely rescued by the loss of P53 [18–20]. In particular, several studies have explored the developmental effects of the loss of *MDM2* or *MDMX*, highlighting that they are highly tissue-specific and, although associated with uncontrolled activation of P53 target genes, the level of alteration of P53 do not correlate to the severity of the observed tissue-specific morphological alterations [21]. Both *MDM2* and *MDMX* are frequently found altered in human cancers (Fig. 1). The *MDM2* gene is overexpressed, mainly by gene amplification, in several solid cancers [22] including in situ and invasive breast adenocarcinomas [23], sarcomas (either common bone and soft tissue forms) [24–26], esophageal cancer [27], and endometrial stromal tumor [28]. It is of interest that, while amplification of the *MDM2* gene is a major mechanism of MDM2 overexpression, MDM2 gene amplification and mutation of *P53* are commonly mutually exclusive events [26]. Moreover, genetic variants of the *MDM2* gene such as single nucleotide polymorphisms affect the basal levels of MDM2 and altering P53 mono- and poly-ubiquitination [29–31]. In the same fashion, *MDMX* can be also found amplified or overexpressed in a number of types of tumors including glioblastomas [32], retinoblastomas [33], as well as sarcomas, and breast cancers [22]. Amplification of *MDM2* and amplification/overexpression of *MDMX* together with alterations of P53 in the context of hematological malignancies are summarized in Table 1.

**Table 1:** Frequency of alterations of *TP53*, *MDM2*, and *MDMX* in relevant hematological neoplasia diseases

Disease	*TP53* mutations	*TP53* 17p deletion	*MDM2* overexpression/trisomy 12	*MDMX* overexpression	References
ALL	13.5 ± 0.7 (459 pt)	4.3 ± 1.2 (1731 pt)	29.3 ± 4.7 (101 pt)	80 (55 pt)	[147–150]
AML	13.5 ± 2.7 (2934 pt)	1.5 ± 0.7 (2398 pt)	49 (189 pt)	10.7 (140 pt)	[150–156]
BL	17.3 ± 9 (218 pt)	10 (28 pt)	13.5 ± 13.4 (109 pt)	21 (30 pt)	[157–159]
CLL	10.2 ± 1.5 (3703 pt)	6.8 ± 1.9 (3523 pt)	13.8 ± 1.8 (3523 pt)	21 (131 pt)	[76, 150, 160–164]
MM	8.2 ± 5.2 (733 pt)	10.2 ± 2.7 (954 pt)	8 (82 pt)	na	[165–170]
NHL	24.4 ± 6.4 (1337 pt)	25 ± 7.1 (75 pt)	15.1 ± 17.2 (851 pt)	na	[171–177]
WM/LPL	7 (30 pt)	8.7 ± 1 (206 pt)	4 ± 1.4 (214 pt)	na	[178–182]

The percentages of abnormalities were calculated after compiling cases reported in literature (total number of patients is indicated in bracket) and are reported as mean ± standard deviation

*ALL* acute lymphoblastic leukemia, *AML* acute myeloid leukemia, *BL* Burkitt lymphoma, *CLL* chronic lymphocytic leukemia, *MM* multiple myeloma, *NHL* non-Hodgkin lymphoma, *WM/LPL* Waldenström macroglobulinemia/lymphoplasmacytic lymphoma, *na* not available

### The MDM2/X axis with P53

In normal conditions, to maintain an appropriate biological outcome, cellular homeostasis is set to preserve low P53 levels and avoid excessive P53 accumulation with a concomitant and unnecessary activation of the P53-mediated pathways. Accumulation and activation of P53 are strictly regulated processes under the control of several regulatory signals and molecular mediators [34, 35]. Intracellular P53 is maintained low mainly thanks to the short half-life of the protein that is subjected to constant proteasome-dependent degradation. This is a reversible process allowing, when necessary, the rapid interruption of the protein degradation with an increase of protein levels to react and respond to specific signals of cellular stress and damage. Ubiquitination-mediated proteasome degradation is the critical process involved in regulating cellular accumulation and activity of P53 [36]. Nonetheless, ubiquitin-independent mechanisms are not excluded [37].

Ubiquitin-mediated protein degradation is a three-step process involving at least the E1 (Ub-activating) enzyme, the E2 (Ub-conjugating) enzyme, and E3 (Ub protein) ligase. A number of E-ligases able to mediate P53 ubiquitination have been identified so far, and the list includes, within the others, molecules such as COP1, CHIP, ARF-BP1, MKRN1, and several members of the TRIM protein family [36, 38–44]. Nonetheless, the best-validated ubiquitin ligase for P53 is MDM2, a protein that exerts its negative regulatory activity at different levels. The binding of the MDM2 amino-terminal domain to P53 is sufficient to inhibit the transcriptional activity of P53 affecting both the P53-mediated cell cycle arrest and the apoptosis functions [45]. Moreover, the presence of a nuclear export signal into MDM2 allows, after the binding of P53, the export from the nucleus to the cytoplasm of the protein complex [46]. This process has a dual effect: the prevention of P53 binding to the DNA precluding its activity as transcription factor and the degradation of P53 by cytoplasmic proteasomes [47–49]. The proteasome-mediated P53 degradation occurs via engagement of the really interesting new gene (RING) domain of MDM2 (i.e. really interesting new gene domain, the one that retains the ligase activity) to the N-terminal transactivation domain of P53, with the transfer of ubiquitin primarily into six lysine residues of the C-regulatory region of P53, the main sites of ubiquitin ligation [50, 51]. However, although MDM2 ubiquitin ligase activity is mandatory for P53 processing, MDM2 can catalyze both mono-ubiquitination (conjugation with an ubiquitin monomer at one or multiple sites) and poly-ubiquitination (conjugation with a polymeric ubiquitin chain) of P53 in a dosage-dependent manner [52]. In particular, in the presence of low levels of MDM2, it is likely that P53 undergoes to MDM2-mediated mono-ubiquitination and cytoplasmic translocation, while in the presence of stressed conditions MDM2 activities are high and there is a preferential poly-ubiquitination of P53 leading to degradation in the nucleus [52].

**Fig. 2:**
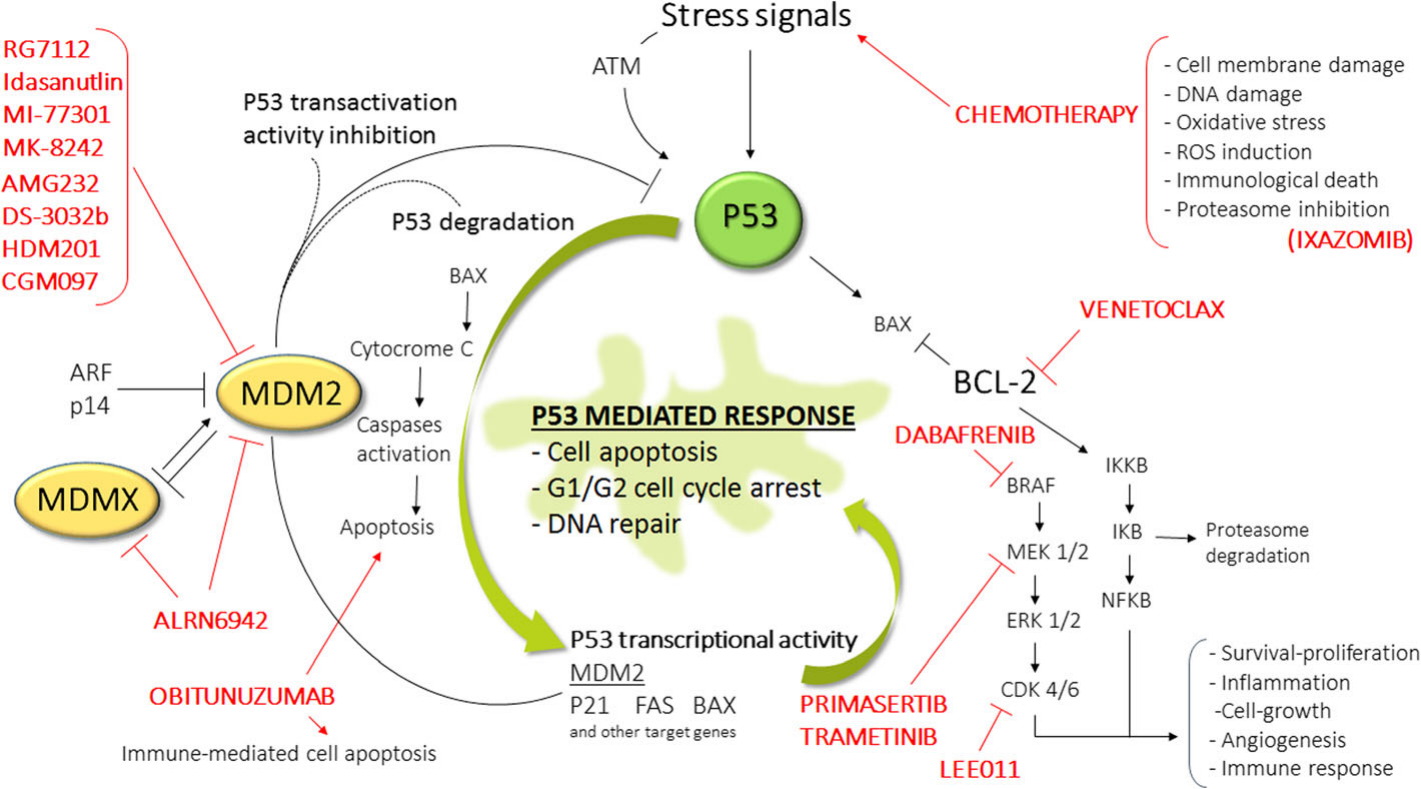
Schematic representation of the autoregulatory loop between P53, MDM2, and MDMX and pharmacological inhibitors. The picture shows the main molecular links between P53, MDM2, and MDMX together with the MDM2 and MDMX inhibitors under clinical evaluation and the key compounds used in clinical studies for combination therapy

As transcriptional factor, P53 binds the promoter region of MDM2 and regulates the protein expression in an autoregulatory feedback loop that is critical in maintaining the appropriate balance between the levels of both MDM2 and P53 proteins (Fig. 2) [53]. Briefly, in normal conditions, P53 acts as transcription factor of the MDM2 gene and induces expression of MDM2 protein that binds P53 and induces in turn its degradation. The overall result is a tight and constant balance of the two proteins’ levels [53]. The autoregulatory loop between P53 and MDM2 crosses with MDMX that is able to interact with both P53 and MDM2. In particular, MDMX binds the N-terminal transactivation domain of P53 and represses P53 transcriptional activity without a direct E3 ligase activity [16], and differently from MDM2, the absence of P53-responsive elements in the MDMX structure makes MDMX not susceptible of P53-mediated regulation. On the other side, the presence of a RING domain allows MDMX to cross-reacts with MDM2 creating RING-mediated heterodimers [54, 55]. In the form of heterodimer with MDMX, MDM2 is more stable and exhibits a higher efficacy in downregulating P53 [56]. The MDMX-mediated regulation of MDM2 and the implications for P53 regulation have been recently addressed [57]. Overall, it is widely accepted that MDMX and MDM2 are two non-overlapping inhibitors of P53 [58], but it has been demonstrated that they can act downregulating P53 through the generation of heterodimers in normal conditions but also through a positive regulation of P53 after DNA damage [59]. In particular, the switch between negative and positive regulators occurs following the phosphorylation on serine residues that changes the conformation of the proteins re-directing the E3 ubiquitin ligase activity of MDM2 away from P53 and toward MDMX and MDM2 itself [59].

Overall, the deep understanding of the MDM2/MDMX-P53 axis achieved in the last years has provided the biological rationale for the design of innovative chemical and synthesis procedures to develop molecules able to inhibit this interaction and restore the antitumor activity of P53. Crystal structure studies of P53 in complex with the MDM proteins have highlighted that three amino acids, Phe19, Trp23, and Leu26, play a key role in the MDM2-P53 interaction, differently from the MDMX-P53 interaction that must be targeted independently from MDM2, opening the doors to the identification of several classes of antitumor agents with different clinical potential [60, 61]. From a clinical perspective, the predominant role of MDM2 and MDMX in regulating P53 through the P53-MDM2/X axis has led to several reports in this field in the last few years; however, suggestions about new compounds and therapeutic combinations are coming from preclinical studies, and novel compounds have recently entered into clinical trials.

In this light, the aim of this review is to give an updated overview of the impact of the newly discovered compounds and to revise and comment the recent suggestions coming from their clinical evaluation as monotherapy or in combination with conventional chemotherapy and innovative therapeutic combinations, with particular attention to their potential for hematological malignancies and pediatric cancers.

### Targeting the MDM2/X-P53 axis for therapeutic purposes

In the past few years, several molecules have been developed in order to release P53 from the control of MDM2 and restore its oncosuppressor activity. This goal has been obtained through different “molecular strategies” aiming at (i) blocking MDM2 expression, (ii) blocking the physical interaction between MDM2 and P53, (iii) modulating the E3 ubiquitin ligase activity of MDM2, and (iv) targeting the MDM2-P53 (protein–protein) complex. Insight on structural studies and chemical/technological procedures involved in the design of successful MDM2/X inhibitors and processes in understanding their potential are beyond of the purposes of the present reviews, and the details can be found in several recent publications [62–64]. Here, we will focus on the compounds that are now under clinical evaluation (and summarized in Table 2) to discuss their clinical potential and the recent results coming from their assessment.

**Table 2:** Clinical trials with MDM2-MDMX inhibitors

Compound	Clinical study	Phase	Status	Clinical trial identifier
RG7112 RO5045337	Extension study to provide treatment to participants who have completed parent studies	1	Active, not recruiting	NCT01677780
	Solid tumors	1	Completed	NCT01164033
	Patients with liposarcoma who are eligible for debulking surgery	1	Completed	NCT01143740
	Soft tissue sarcoma: treatment in combination with doxorubicin	1	Completed	NCT01605526
	AML, ALL, CML in blast phase, refractory CLL/SCLL	1	Completed	NCT00623870
	Advanced solid tumors including lymphoma	1	Completed	NCT00559533
	AML: treatment combination with cytarabine	1	Completed	NCT01635296
RG7388 RO5503781 Idasanutlin	Essential thrombocythemia and polycythemia vera	1	Recruiting	NCT02407080
	Solid tumors: determination of excretion balance, pharmacokinetics, metabolism and bioavailability	1	Active, not recruiting	NCT02828930
	Multiple myeloma: combinations with ixazomib citrate and dexamethasone	1/2	Recruiting	NCT02633059
	R/R AML: combination with cytarabine	3	Recruiting	NCT02545283
	In R/R FL in combination with obinutuzumab and in DLBCL combination with rituximab	1b/2	Recruiting	NCT02624986
	R/R AML patients not eligible for cytotoxic therapy: combination with venetoclax	1/2	Recruiting	NCT02670044
	Prostate cancer who haven’t had docetaxel: Idasanutlin with abiraterone or enzalutamide	1/2	Open	CRUKE/12/032
	Solid tumors: effect of posaconazole on pharmacokinetics, bioavailability of new formulations	1	Completed	NCT01901172
	AML: combination with cytarabine	1	Completed	NCT01773408
	Advanced malignancies except leukemia	1	Completed	NCT01462175
SAR405838 MI-77301	Advanced cancer	1	Active not recruiting	NCT01636479
	Advanced cancer (solid tumors): combination with pimasertib	1	Completed	NCT01985191
MK-8242 SCH 900242	Advanced solid tumors: study of safety and pharmacokinetics	1	Completed	NCT01463696
	AML: alone and in combination with cytarabine	1	Completed	NCT01451437
AMG232	R/R AML: combination with trametinib	1b	Recruiting	NCT02016729
	R/R multiple myeloma: combination with carfilzomib, lenalidomide, dexamethasone	1	Not yet recruiting	NCT03031730
	Advanced solid tumors or multiple myeloma	1	Active not recruiting	NCT01723020
	Metastatic cutaneous melanoma: combination with trametinib and dabrafenib or trametinib	1b/2a	Recruiting	NCT02110355
DS-3032b	Relapsed and/or refractory multiple myeloma	1	Recruiting	NCT02579824
	Hematological malignancies: AML, ALL, CML and myelodysplastic syndrome	1	Recruiting	NCT02319369
	Advanced solid tumors or lymphomas	1	Recruiting	NCT01877382
HDM201	Liposarcoma in combination with LEE011	1b/2	Recruiting	NCT02343172
	Advanced solid and hematological tumors with wt-TP53	1	Recruiting	NCT02143635
	Neuroblastoma: to match genomic aberrations at time of relapse to designed combined therapies	1	Recruiting	NCT02780128
CGM097	Advanced solid tumors with wt-TP53	1	Active not recruiting	NCT01760525
ALRN-6924	AML or advanced myelodysplastic syndrome with wt-TP53: alone or in combination with cytarabine	1/1b	Recruiting	NCT02909972
	Advanced solid tumors or lymphomas with wt-TP53	1/2a	Recruiting	NCT02264613

Data from ClinicalTrials.gov. National Library of Medicine: http://www.clinicaltrials.gov and Cancer Research UK: http://www.cancerresearchuk.org. Accessed April 2017

*R/R* relapsed/refractory, *FL* follicular lymphoma, *DLBCL* diffuse large B cell lymphoma

### The cis-imidazolines “Nutlins” compounds: RG7112 and RG7388 (Idasanutlin)

The RG7112 (RO5045337) molecule from Roche has been the first MDM2 inhibitor undergoing clinical evaluation. This molecule derives from the optimization of the original molecules belonging to the Nutlins, a group of small molecules (Nutlin-1, Nutlin-2, Nutlin-3) acting by preventing the binding of MDM2 to P53. At the structural levels, the Nutlins are cis-imidazoline analogs discovered by Vassilev and colleagues in 2004 as compounds able to bind MDM2 in the P53-binding pocket with subsequent P53 accumulation, P53 pathways activation with the induction of cell cycle arrest, and apoptosis and growth inhibition in cancer cells and human tumor-xenografted nude mice [65]. In addition to P53 activation, it has been also reported that Nutlin-3a (the most active and potent enantiomer of the Nutlins’ family) was able to induce P53-independent mechanisms of cell death by enhancing the stability of P73, a member of the P53 family which is also regulated by MDM2 and that shows pro-apoptotic activities [66]. Actually, the interaction with P73 is not the only P53-independent effect of MDM2, since additional regulatory targets of MDM2 have been reported such as pRb [67] and E2F/DP [68]. In fact, it has been shown that antagonism of MDM2 by Nutlin-3a in cells with mutant P53 enhances chemo-sensitivity in an E2F1-dependent fashion, providing a therapeutic advantage also in tumors characterized by mutant P53 in combination with chemotherapy [69]. In terms of preclinical evaluation, it has been widely demonstrated in both in vitro and in vivo tumors models that Nutlin-3a is a powerful antitumor agent able to target different types of solid tumors, such as breast cancer, melanoma, retinoblastoma, prostate cancer, and lymphoma, as well as hematological malignancies. In particular, in the context of melanoma, important results have been achieved by using Nutlin-3 both as single treatment, revealing a complete inhibition of tumor cells invasiveness [70], and in combination with other molecules, such as inhibitors of Aurora A kinase that are synergized with Nutlin-3 by promoting immune-mediated tumor clearance in patient-derived xenograft models [71]. Also in the context of breast cancer in recent years, the use of Nutlin-3 was preclinically validated in combination with (i) carboplatin, in a humanized orthotropic breast-to-lung metastatic model [72] and (ii) paclitaxel, against aggressive diseases such as triple-negative and Numb-deficient human breast cancers [73, 74]. In particular, the combination Nutlin-3 plus paclitaxel in patient-derived xenograft (PDX) models showed persistent tumor growth inhibition and prevention of cancer stem-cell-driven tumor relapse after removal of chemotherapy [74]. In the context of hematological malignancies, our group has significantly contributed to the demonstration and characterization of the potential of Nutlin-3 in preclinical models based on established cell lines and patient-derived cells of acute myeloid leukemia (AML) and of chronic lymphocytic leukemia (CLL). In these settings, the efficacy of Nutlin-3 has been related to activation of P53 apoptotic pathway [75, 76] and modulation of a specific panel of genes and proteins related to cell survival [77–80], apoptosis [81, 82], and cell cycle progression [83]. Other groups validated the cytotoxic preclinical effects of Nutlin-3 in different hematological malignancies. In particular, significant results were obtained in the field of acute lymphoblastic leukemia (ALL), demonstrating the efficacy of the activation of P53 pathway and subsequent cell cycle block and apoptosis induction in aggressive childhood leukemia cells in vitro and in mice xenograft [84, 85] as well as reduction of cell viability, involving also autophagy, in adult ALL samples [86]. Our group has also significantly contributed to the demonstration of a synergistic activity of Nutlin-3 in different leukemic models, irrespectively of the P53 status: (i) with the multi-kinase inhibitors Dasatinib and Sorafenib in B cell chronic lymphocytic leukemia (B-CLL) and AML models via inhibition of the Akt pathway by inducing apoptosis/autophagy [87, 88]; (ii) with nanoparticles engineered with rituximab thus targeting CD20^+^ cells enhancing antibody-dependent cellular cytotoxicity and increasing survival in B-leukemic xenografted mice [89, 90]; (iii) with sodium dichloroacetate via increased expression of the P53-target genes *MDM2*, *PUMA*, *TIGAR*, and *CDKN1A* [91]; and (iv) with ibrutinib via inhibition of the BCR signaling and MAPK/PI3K pro-survival pathways [92]. The functional activation of P53 pathway after ex vivo Nutlin-3 treatment was proposed by Pozzo et al. as assay to detect P53 dysfunction in B-CLL patient samples [93]. The first published results on the clinical potential of RG7112 have been obtained in a trial of the European Community (EudraCT number: 2009-015522-10) investigating the pharmacodynamics of RG7112 in patients with MDM2-amplified liposarcoma [94]. Although reporting an overall feasibility of RG7112-mediated inhibition of MDM2 and P53 activation in this type of tumor in vivo, the trial highlighted several clinical adverse events related to the drug treatment including hematological toxicity, making the long-term treatment with RG7112 a challenge [94]. A phase I study on patients with advanced solid tumors has assessed the dose and treatments protocols showing that although RG7112 was generally well tolerated, the more effective schedule in terms of adequate P53 tumor activation was anyhow associated to increased hematological toxicities [95]. RG7112 has been clinically evaluated also in a phase I study clinical study involving different types of hematological malignancies including relapsed/refractory AML, ALL, chronic myeloid leukemia (CML) as well as CLL, and small cell lymphocytic leukemia (sCLL). RG7112 showed sufficient clinical activity to lead to P53 stabilization and transcriptional activation of P53 target genes in extremely poor prognosis (relapsed/refractory) AML patients and in CLL/sCLL patients [96]. In terms of potential molecular biomarkers, treatment with RG7112 led to increased expression of several P53 target genes including *BAX*, *PUMA*, *FDXR*, *MDM2*, *ZMAT3*, *FAS*, *TNFRSF10B*, *CDKN1A*, and *TP53NP1* in circulating P53 wild-type leukemic cells following different kinetics of activation [96]. In AML, RG7112 administration showed clinical activity as monotherapy, particularly in relapsed/refractory AML with some patient reaching complete remission and hematopoietic recovery allowing subsequent transplantation [96], as well as by combining RG7112 with cytarabine, suggesting that combinations with other therapeutic agents might result in a synergic effect [97].

The evidence of hematotoxicity following treatment with RG7112 was not surprising since MDM2 plays a role in normal hematopoiesis. Indeed, preclinical studies highlighted that MDM2 antagonists induce major hematopoietic defects and inhibition of megakaryopoiesis that may account for the significant marrow suppression of both leukemic and normal progenitors in patients following the treatment [98]. The high doses required to obtain a clinical effect together with the impact of toxicities and complications (i.e., neutropenia, thrombocytopenia including sepsis and hemorrhage) and gastrointestinal toxicity due to the loss of enterocytes attributable to RG7112 administration have prompted the development of a more potent and selective compound of the Nutlins’ family named RG7388 (RO5503781) known as idasanutlin [99]. Idasanutlin is a second-generation MDM2 inhibitor able to induce the expected biological effects at concentrations that are significantly lower than those required by RG7112, both in in vitro and in vivo experimental models [99]. The higher efficacy and the less degree of side effects compared to the original compound have therefore speed up the setting of several clinical trials (Table 2). A recent phase Ib study on relapsed-refractory AML patients has assessed safety and pharmacokinetics of escalating doses of idasanutlin as single agent or in combination with cytarabine, showing a well tolerability of the treatment coupled with clinical response and with the possibility of achieve a good control of the gastrointestinal complications [100]. Moreover, analysis of MDM2 protein expression showed that the levels of MDM2 expression in leukemic blasts are associated to idasanutlin clinical response and might therefore represent a useful biomarker to identify those AML patients who likely will have advantage from idasanutlin treatment [100]. Different idasanutlin-based combined approaches are under evaluation at the clinical level. Among these, the combinations with obinutuzumab and venetoclax might potentially have a significant impact in the context of hematological malignancies. Obinutuzumab is a novel glycoengineered type II anti-C20 monoclonal antibody characterized by enhanced antibody-dependent cellular cytotoxicity and direct cell death compared to rituximab and representing therefore a significant clinical improvement for the treatment of B cell malignancies [101]. Recently, the antitumor potential of the therapeutic combination based on the caspase-independent cell death and antibody-dependent cellular cytotoxicity triggered by obinutuzumab together with the caspase-dependent apoptosis mediated by idasanutlin has been evaluated in in vitro and in vivo models of lymphoma, reporting an enhanced P53 wild-type cell death without signs of reciprocal interference between the two pharmacological compounds [102]. Venetoclax (ABT-199) is a selective oral small molecular BCL-2 inhibitor that has been tested at the preclinical level in combination with idasanutlin in P53 wild-type AML models, showing indeed a synergistic antitumor activity compared with the respective single-agent treatments [103]. Therefore, these studies provided the proof of concept for the clinical evaluation of such therapeutic combinations.

### The spirooxindole MI-77301 (SAR405838)

The small-molecule MI-77301 from Sanofi is a spirooxindole-based compound that mimics the three P53 key aminoacidic residues but shows also the ability of establishing additional interaction with an overall a higher binding affinity. After optimization of the two phenyl rings, the newly derived molecule was characterized by an improved affinity to MDM2 leading to an optimized protein binding and enhanced biological activities together with improved in vivo pharmacokinetic features. In particular, the preclinical evaluation of the antitumor activity of MI-77301, also referred as SAR405838, revealed a good chemical stability of the compound and the capacity of inducing cell growth inhibition up to cell cycle arrest and/or apoptosis in a P53-dependent manner in several tumor cell lines (i.e., osteosarcoma, acute leukemia, prostate and colon cancer cells) [104]. In in vivo xenograft models, SAR405838 was able to induce a significant antitumor activity in different types of tumors characterized by P53 wild-type status but lacking MDM2 amplification and a complete and persistent tumor regression in 100% of mice bearing osteosarcoma cancer cells with wild-type P53 and amplified MDM2 [104]. At the molecular level, the pharmacological treatment was able to induce upregulation of MDM2 and P21 in all cancer cell lines and PUMA expression was correlated with apoptosis induction and tumor regression suggesting a direct role in mediating SAR405838 biological effects [104]. A clinical study evaluated the combined treatment of SAR405838 with pimasertib (a small-molecule inhibitor of MEK1 and MEK2) in patients with advanced solid cancer. Although the study is terminated, no results have been published so far. Results from a second clinical trial assessing SAR405838 safety, tolerability, pharmacokinetics, and biological activity in patients with advanced cancer, including solid tumors and lymphoma, for which no further effective standard treatment is available, have been recently published. The pharmacological treatment resulted to be acceptable in terms of safety, and a dose-dependent MIC-1 modulation following P53 pathway activation was observed suggesting that, although a limited activity was observed with the single-agent schedule, SAR405838 might have a potential in combination regimens [105].

### The MK-8242 compound (SCH-900242)

MK-8242, also known as SCH-900242, is a compound from Merck pharmaceutic described as a potent, orally bioavailable, small-molecule inhibitor of the MDM2-P53 protein–protein interaction able to induce growth arrest and cell death at IC50 value as low as 20 nM [106]. Two phase I clinical trials have been launched by Merck to evaluate MK-8242 alone in patients with advanced solid tumors and MK-8242 either alone or in combination with cytarabine in AML patients (Table 2). In the context of AML, the study aimed at evaluating safety, tolerability, and pharmacokinetics in adult patients with refractory or recurrent AML together with the evaluation of the response rate and duration of response after treatment [106]. The results of the study highlighted adequate safety and no significant adverse effects, even though the maximum dose tolerated and the recommended phase 2 dose were not established for the better tolerated 7-day on/14-day off dosing schedule as well as the single-agent tolerance since doses above 300 mg (administered twice a day) were not tested [106]. In terms of toxicity, one patient reported a grade 3, drug-related adverse event of atrial fibrillation while the most common side effects were in line with those described with other MDM2 inhibitors including in particular gastrointestinal and hematological adverse events that are dose dependent and mostly less than grade 3 resulting therefore overall well handily. In terms of efficacy assessment, the single-agent clinical effectiveness was limited, probably for the advanced disease state of the patients and the limited number of treatments performed. Moreover, although one of the clinical aims of the study was the evaluation of the combined treatment with chemotherapy (cytarabine), the study terminated before the complete enrollment of this arm of the study. The results of the second study performed in patients with advanced/refractory solid tumors characterized by wild-type P53 status have been recently reported [107]. The 47 patients that received MK-8242 monotherapy (doses ranging from 60 to 500 mg) developed gastrointestinal/hematologic toxicity in line with the previous study. Doses above 300 mg were able to activate P53 pathway, as demonstrated by the expression of the P53 target gene pleckstrin homology-like domain family A member 3 (*PHLD3A*) which is a biomarker of P53 transcriptional activity, supporting the designation of 400 mg as the recommended phase II dose. The clinical activity was however less impressive than expected showing three patients with liposarcoma with a partial response, 31 patients with stable disease, and eight with progressive disease for a total of 27 patients with liposarcoma with a median progression-free survival of 237 days [107]. These partially satisfying results could be due to previous chemotherapy that might have selected more resistant tumors, and perhaps the achieved P53 activation might have been insufficient to induce significant cell death [107]. Nonetheless, the median progression-free survival appears encouraging, in particular in patients with liposarcoma.

### New compounds recently entered in clinical evaluation

In the last few years, additional compounds and small-molecule inhibitors of MDM2/X have entered in clinical evaluation.

AMG232 is a piperidinone-derived compound from Amgen acting as a potent and selective inhibitor of the MDM2−P53 interaction with notable pharmacokinetic properties and in vivo antitumor activity in xenograft models [108]. The preclinical pharmacological characterization showed a high potential of this MDM2 inhibitor compared to other MDM2 inhibitors including RG7112, SAR299155, and RG7388, in blocking tumor cell proliferation and inducing tumor cell apoptosis in a large panel of tumor cell lines in a P53-dependent manner [109]. As expected, the degree of response to the treatment was variable within the different cell lines both in vitro and in vivo, but interestingly showing antitumor activity also in P53 wild-type tumors carrying different genetic aberrations such as KRAS-mutant and BRAF-mutant. Treatment was associated with P53 stabilization and induction of MDM2, P21, and PUMA expression [109]. Moreover, there was an improved antitumor activity associated with P53 increase and p21 induction when used in combination with cytotoxic agents [109]. In addition, in combination with radiation therapy, the compound showed a more potent antitumor activity coupled to antiangiogenesis potential compared to the single treatments, leading to accumulation of several molecular mediators of senescence/autophagy/apoptosis such as *FoxM1*, *ULK-1*, *DRAM*, and *BAX* [110]. AMG232 is currently under clinical evaluation for the treatment of solid tumors, melanoma, myeloma, and AML leukemia as single treatment or in combination therapies (Table 2).

The CGM097 compound is a substituted dihydroisoquinolinone derivative from Novartis designed to mimic three key hydrophobic interactions made by P53 residues with Phe19, Trp23, and Leu26 in the MDM2 pocket and acting as potent and selective MDM2 inhibitor [111]. The compound is in phase I study as single agent (dose escalation and a dose expansion) in patients with advanced solid tumors characterized by wild-type P53 that undergo standard therapies, but have progressed and in patients with no standard therapy available (Table 2). In the context of hematological malignancies, CGM097 has been evaluated in vitro, showing a potent and selective effect of inhibition of cell proliferation/viability of wild-type P53 primary AML cells and AML cell lines and in vivo antitumor effects in xenograft models, providing the rational for the testing of CGM097 in AML patients [112]. Finally, CGM097 was preclinically evaluated in 24 B cell acute lymphoblastic leukemia patient-derived xenograft (PDX) including hypodiploid, near haploid, mixed lineage leukemia (MLL)-rearranged, CRLF2-rearranged, and BCR-ABL models of the PDX repository (PRoXe; www.proxe.org) showing the ability to induce a significantly improved survival of tumor-bearing mice [113, 114].

The DS3032b compound is an inhibitor of the P53-MDM2 interaction developed by Daiichi Sankyo that has reached the clinical assessment in 2013, and it is now under evaluation in three studies in patients affected by different types of tumors including AML, ALL, CML, MDS (id: *NCT02319369*), advanced solid tumors or lymphomas (id: *NCT01877382*), and relapsed/refractory multiple myeloma patients (id: *NCT02579824*). Recent preliminary results have been presented at the Annual American Society of Clinical Oncology in 2016 on data collected from 31 of 34 patients with solid P53 wild-type tumors (mainly liposarcoma) enrolled in part 1 study. In terms of drug-related adverse effects, gastrointestinal and hematological events were reported with thrombocytopenia or with neutropenia. In terms of efficacy, none of the patients under evaluation had an objective response but the 77% of patients had stable disease with the better outcome in tumors with aberrant MDM2 signaling and wild-type P53 [115]. In December 2016, at the 58th Annual Meeting of the American Society of Hematology Daiichi Sankyo has also announced preliminary safety and efficacy data from the phase I study of DS-3032b in the treatment of hematological malignancies. The dose escalation study in 38 patients enrolled with relapsed/refractory AML or high-risk myelodisplastic syndrome (MDS) has established the maximum tolerated dose at 160 mg once a day for 21 days in a 28-day cycle [116]. In terms of clinical efficacy, the authors report a reduction of bone marrow blasts at the end of the first cycle in 15 out of 38 patients and complete remission in two patients with relapsed/refractory AML [116]. One patient with high-risk MDS achieved marrow complete remission with platelet improvement for 4 months. The three patients (two with AML and one with MDS) showing complete response developed *TP53* gene mutation while receiving treatment, suggesting that a combination therapy approach might be more appropriate [116].

HDM201 from Novartis is an imidazopyrrolidinone scaffold-based inhibitor of the P53-MDM2 protein–protein interaction with superior characteristics in terms of in vitro activity/selectivity and of in vivo features of oral bioavailability, pharmacokinetic, and pharmacodynamic profiles as assessed in animals [117, 118]. In particular, the optimized interactions of HDM201 with MDM2 protein are responsible for the increased stabilization of the complex leading to a higher potency of the molecule [119]. In xenograft tumor models, HDM201administration following either a daily low-dose schedule or once at a high-dose schedule induced a differential response; although, the single high-dose schedule lead to rapid and significant induction of P53-dependent PUMA expression and apoptosis together with robust and sustained tumor regression, though the two regimens had an overall comparable long-term efficacy [120]. There are three ongoing clinical studies aimed to assess and compare different schedules of HDM201 in patients with advanced P53 wild-type tumors (Table 2). Some preliminary results obtained from these trials have been recently reported [121]. The 74 patients receiving HDM201 following two regimens reported common grade 3/4 adverse events including anemia, neutropenia, and thrombocytopenia. Gastrointestinal toxicity was common but not dose limiting (mainly nausea) while hematological toxicity appears to be regimen-dependent and at late onset. Clinical benefit was observed but no further details are available so far [121].

Stapled peptides (i.e., stabilized alpha-helical peptides) are currently rising interest as strategy to target protein–protein interactions. At the chemical level, by virtue of the role of the alpha helix in binding and modulating protein–protein interactions, there is the possibility of “mimicking” the α-helices at the binding interface of two proteins to competitively inhibit their contact [122]. As far as the interaction between P53 and MDM2/MDMX is concern, several reports suggest that MDM2 antagonists might not be effective in tumors overexpressing MDMX, highlighting the potential of a concomitant and simultaneous targeting of the two oncosuppressor that have led to the development of “dual inhibitors” of MDM2 and MDMX for cancer therapy [123, 124]. In this light, the stapled peptide ALRN-6924 from Aileron Therapeutics is the first clinical drug candidate that binds, in an equipotent way, and inhibits the two P53 suppressor proteins MDM2 and MDMX. This molecule is now under clinical evaluation in two studies with advanced hematological (id: NCT02909972) and solid malignancies (id: NCT02264613) characterized by wild-type P53 status.

### Perspectives for pediatric malignancies

The progresses made by recent medicine have led to the achievement of a high rate of overall survival across pediatric cancer patients. The reported 5-year survival is nearly the 80% for many childhood tumors, and it reaches the 90% in the case of pediatric ALL, with a mortality persistently decreasing [125, 126]. However, this outstanding result has left somehow pediatric cancers excluded from the assessment of newly derived therapeutic molecules. The gap between adult and pediatric tumors consideration is well described by the evidence that, although from 1948 to 2003 the FDA approved the use of 120 new cancer drugs, only 30 have been used in pediatric patients [127]. Anyhow, there are still childhood cancers difficult to treat and, as in the case of pediatric ALL, the occurrence of genetic alterations and the development of relapse disease make the clinical outcome less favorable by using the current standard care, pressing the need of new therapeutic options [128]. In addition, therapy-related late adverse effects in cancer survivors, ranging from infertility and cardiotoxicity up to second cancers development, are becoming key issues for therapy-related risk evaluation and therapy decision [129]. With the aim to establish an international platform in order to identify more effective treatments for children with cancer, a pediatric preclinical testing program (PPTC) has been established. Its aim is to provide reliable preclinical in vivo data using genomically characterized patient-derived xenograft lines in order to identify agents to move forward in pediatric clinical trials (NCI PPTC, www.ncipptc.org). The panel of models accessible through the Consortium for testing drug candidates includes tumor cell lines and xenograft models with different features among which most are patient-derived xenografts (PDX) models obtained by direct implant of tumors in mice [130]. A key feature of pediatric cancers is the low frequency of P53 mutations compared to adult tumors, and it is therefore not surprising that the Nutlins compounds exhibited both in vitro and in vivo antitumor activity against several types of pediatric tumors including solid and hematological malignancies [131]. In the same fashion, the preliminary evaluation of the Nutlin-3-derived compound RG7112, tested in vitro against a panel of 23 pediatric cancer cell lines and in vivo in xenograft models, reported regressions in solid tumors from different histotypes including medulloblastoma, alveolar rhabdomyosarcoma, Wilms, rhabdoid, and Ewing sarcoma xenografts [132]. Moreover, with regard to hematological malignancies, RG7112 showed a significant antitumor activity against ALL xenografts including B cell precursor acute lymphoblastic leukemia (BCP-ALL), T cell acute lymphoblastic leukemia (T-ALL), and MLL-ALL with the most impressive effect reported on infant MLL-rearranged xenograft when RG7112 was used either alone or in combination with established drugs such as vincristine, dexamethasone, and l-asparaginase [85, 132]. In line with previous evidence, in these models, the RG7112-mediated effects led to the induction of P53-dependent cell cycle arrest and apoptosis with the activation of pro-apoptotic targets, such as PUMA, and downregulation of the anti-apoptotic protein surviving supporting the use of this Nutlin-derived molecule in pediatric cancer management [85]. In the pediatric context, while carcinomas are very rare (1.5% of malignancies), the embryonal tumors represent more than one-fourth of all malignant diseases, with neuroblastoma being the most common extracranial solid tumor of childhood [133]. Although patients are generally responsive to treatments, children with high-risk neuroblastoma have also a high risk of relapse and show high rate of mortality. Since P53 is usually unaltered in this type of tumor and genetic alterations of *TP53* are rare (in contrast with aberrations of *MDM2* that are instead quite common), the use of MDM2 inhibitors to target the P14(ARF)-MDM2-TP53 key axis in neuroblastoma is an appealing therapeutic strategy [134]. In this line, it has been shown that Nutlin 3 is able to induce P53 accumulation with concomitant decrease of proliferation and increased apoptosis in neuroblastoma cells when used alone and in synergy with chemotherapeutic drugs [135]. Moreover, synergistic antitumor activity in neuroblastoma has been demonstrated in different therapeutic combinations such as the one based on Nutlin-3 plus bevacizumab to enhance apoptosis and target tumor angiogenesis [136]. Of note, a sensitizing activity to genotoxic drugs of Nutlin 3 has also been reported in P53-null neuroblastoma cells via upregulation of TAp73 and activation of E2F1, highlighting the P53-independent mechanisms of actions of this inhibitor [137]. The potential therapeutic benefit for neuroblastoma has been evaluated in phase II-like trials with recent MDM2 inhibitors that have entered in clinical evaluation for adult cancers. In this line, Lu and colleagues have reported a P53-mediated apoptotic effect of MI-773 in a P53 wild-type neuroblastoma model with stabilizing effect on P53, both in vitro showing similar efficacy as RG7388 and Nutlin-3 and in an in vivo orthotropic model when the compound was used as single agent. Moreover, when used in combination with doxorubicin, there was a significant a sensitizing activity toward chemotherapy supporting the rational of combination therapies to overcome chemo-resistance [138]. In the context of the PPTP, a second phase II-like trial has been performed to test the potential of MK-8242 as single agent in several tumor models [139]. Kang and co-workers reported sensitivity of different P53 wild-type pediatric tumor cell lines to MK-8242 treatment with an IC50 that was around sixfold lower than the one reported for RG7112 in the same tumor models. The antitumor activity evaluated in vivo highlighted a differential response among the different P53 wild-type solid tumor xenografts, with an overall delay (twofold or greater) in time to event in the treated mice compared to controls, with the exception of the osteosarcoma xenografts that were indeed characterized by a very low P53 expression [139]. The in vivo evaluation of a hematological panel highlighted MLL-rearranged lines as the best responders to MK-8242 treatment, while the ALL-non-MLL models showed only partial responses with no complete remission [139]. Finally, results from a randomized phase II-like trial assessing the MDM2 inhibitor CGM097 in a large and well-characterized leukemia and lymphoma PDX repository (Public Repository of Xenografts, PRoXe; www.proxe.org) have been recently published by Townsend and colleagues [140]. In their work, the authors aimed at testing CGM097 in mice xenografted with adult and pediatric P53 wild-type B-ALL. Overall, treatment with CGM097 led to survival benefit only on wild-type P53 PDX with an increased median survival of 44 days compared to controls; although, heterogeneity of response to CGM097 was observed within the different xenograft models. This study provides the proof of concept for the clinical testing of CGM097 in pediatric B-ALL patients including those that received previous extensive chemotherapeutic treatments [140].

## Conclusions

The use of MDM2/X pharmacological inhibitors to activate the P53 pathway and challenge cancer is an appealing and fruitful therapeutic strategy, particularly for the management of hematological malignancies that show low levels of *TP53* mutations. The ongoing clinical trials are testing mainly inhibitors of MDM2 while chemical compounds targeting MDMX did not advanced to the clinical phase yet. However, there is great effort at the preclinical levels to design successful MDMX inhibitors and there are great expectations on the results of the clinical study with the first double MDM2-MDMX inhibitor ALRN-6924. Overall, the increasing number of newly derived compounds recently entered in clinical trials and the preliminary results coming from these studies are encouraging. As for many drugs, also this class of compounds will definitely benefit of the combination with a second pharmacological strategy, ranging from conventional cytotoxic chemotherapy up to novel small-molecule inhibitors (Fig. 2), allowing a synergic effect in targeting both the P53 wild-type cancer cells as well as the P53-mutated clones. However, several aspects need to be handled in the future studies. The drug-related toxicity is one of the main issue since the simple adjustment of schedule treatments does not appear sufficient to achieve an adequate control of the gastrointestinal, hematologic, and cardiac adverse effects reported when these inhibitors are used either as monotherapy or in combination with standard chemotherapeutic drugs that might “synergize” also in this aspect [98]. An attractive option could be the “cyclotherapy,” designed to perform a conditioning treatment with low doses of P53 activators to induce cell cycle arrest in normal cells with a cytostatic effect that will be protected from the toxicity of conventional drugs targeting the S/M phases of the cell cycle [141]. Another concern is the development of drug resistance that may impair the clinical potential of these compounds. Resistance to treatment occurs through the development of P53 mutant-clones and as result of other molecular defects and/or altered expression of molecules, such as MDMX, that can confer resistance to MDM2 inhibitors. Hoffman-Luca and colleagues have recently addressed the mechanisms of acquired resistance focusing on the MDM2 inhibitor SAR405838. By using an osteosarcoma cell line (MDM2 amplified and *TP53* wild-type) the authors highlighted a difference between the resistance acquired in vitro and the one acquired in in vivo xenograft models [142]. In fact, while the in vitro pharmacological treatment led to the selection of resistant sublines characterized by the development of P53 mutations in the DNA binding domain, the resistant xenografted tumors regrown after drug treatment showed only a partial loss of ex vivo sensitivity to the compound and retained wild-type *TP53*. The different responses and the different mechanisms of acquired resistance in the two models have obviously important implications in terms of drug development and preclinical assessment. The key point appears to be the “pressure” of the drug that is different in the two models: steady and persistent in vitro while variable in vivo with pharmacokinetic-dependent fluctuations. As expected in the first model, the tumor cells need to develop inactivating *TP53* mutations to survive, while in vivo structural mutations might be sufficient to confer the required advantage to the cells to survive [142]. The evidence of acquisition of somatic *TP53* mutations and selection of mutated clones is in line with the preclinical results obtained with other MDM2 inhibitors [143, 144] that provide insight into the mechanisms of acquired resistance useful also to assess the potential of combined/sequential therapies between MDM2 inhibitors and other small molecules such ad Bcl-2 inhibitors [145]. The demonstration of the onset of pharmacological resistance to an MDM2 inhibitor at the clinical level has been recently reported by Jung and colleagues [146]. Data were collected from the phase I trial (NCT01636479) with patients affected by de-differentiated/MDM2 amplified liposarcoma under SAR405838 treatment [105]. *TP53* mutations appeared during treatment as shown by cell-free DNA analysis and the frequency of mutations increased during the time with a correlation with tumor mass [146]. In conclusion, in the next future, the deep elucidation of the mechanisms underlying the onset of acquired resistance in preclinical models and in long-term follow-up-treated patients will be crucial. In the pediatric context, the phase II-like trials that mimic human randomized clinical trials definitely represent a critical step to gain insight on the potential of MDM2/X inhibitors. These preclinical platforms have already allowed the evaluation of the antitumor activity of several compounds. In addition, they have highlighted key aspects that need to be addressed in the next future including the gap between in vitro activity and in vivo efficacy and the high doses required in the xenograft models that would imply very high doses in patients [140]. Finally, a personalized approach would definitely optimized the use of this therapeutic strategy. To this end, the *TP53/MDM2* and *MDMX* status need to be coupled with new biomarkers that might help in selecting those patients that will benefit of such therapeutic approaches either as monotherapy or as combination treatments.

## Abbreviations

ALL: Acute lymphoblastic leukemia
AML: Acute myeloid leukemia
B-CLL: B cell chronic lymphocytic leukemia
BCP-ALL: B cell precursor acute lymphoblastic leukemia
CLL: Chronic lymphocytic leukemia
CML: Chronic myeloid leukemia
MDM2: Murine double minute-2
MDMX: Murine double minute-X
MDS: Myelodysplastic syndrome
MLL: Mixed lineage leukemia
PDX: Patient-derived xenograft
PHLDA3: Pleckstrin homology-like domain, family A, member 3
PPTP: Pediatric preclinical testing program
PRoXe: Public repository of xenografts
RING: Really interesting new gene
sCLL: Small cell lymphocytic leukemia
T-ALL: T cell acute lymphoblastic leukemia
Ub: Ubiquitin
